# Reply to Dr. Barry's Remarks on the Gibraltar Epidemic of 1828

**Published:** 1831-05

**Authors:** Hugh Fraser

**Affiliations:** late Surgeon of the Civil Hospital, Gibraltar.


### GIBRALTAR EPIDEMIC.

*Reply to* Dr. Barry's *Remarks on the Gibraltar Epidemic of* 1828. By Hugh Fraser, Esq., late Surgeon of the Civil Hospital, Gibraltar.

(Concluded from page 318.)

Here terminate the points at issue relating to the contagious or non-contagious nature of the Gibraltar epidemic fever. I think, that the public are now in possession of sufficient materials to enable them to judge on which side truth is most likely to be discovered; and they will probably not feel less indignant than I did, in my first reply to Dr. Barry; or less indignant than many at Gibraltar have long felt, at the manner in which the simplest facts have been moulded to suit particular views and purposes: "Auri sacra fames, quid non—." This would be the place to animadvert, as it deserved, upon a singular effusion of wit* had recourse to by Dr. Barry, and I will venture to say never before attempted on so grave and momentous a subject; but the contrition expressed by the Doctor, in your Journal for February last, induces me to withhold any farther observations on this part of his Rejoinder than saying, that I shall suffer him to adjust his position with his professional brethren, and the public, in the manner he may deem most convenient. He seems to have speculated upon finding some people disposed to agree with him, that the subject of yellow fever was a jocular one, exciting to merriment; so much so that, in truth, as he tells us in your Journal, just quoted, his paper would have been "unreadable without a little raillery;" the public, however, will, I am satisfied, at least perceive that, in making his excuses, he has not the smallest claim to appropriate to himself the "ridentem discere *verum*" of his quotation.

In opposition to my statement as to Dr. Barry having had but few opportunities of seeing the disease, and that he was therefore incompetent to speak *en maître* of it, either as to symptoms, treatment, or otherwise; he admits, that he did not arrive in the bay of Gibraltar until the 8th of November, very late in the epidemic season, as I have stated; did not *land* for *two days* after his arrival, as I have not stated; "observed the general localities of the place," as I have stated, as well as proved, I think, that he had written upon them very amply and properly; "saw and conversed with most of the civil practitioners:" the civil practitioners, Dr. Barry! We have precisely come to the point: pray, Dr. Barry, why did you not go, as the gentlemen of the French commission did, *immediately* on their arrival in the garrison, to the military hospital, which some, perhaps, may think was your place, and where cases of yellow fever were to be seen? For my part, I think that you had a good enough reason: you were, let me tell all who would ask the question, usefully, and very properly employed on the Neutral ground, in drawing up your lengthened observations on the *local origin*† of the "Bulam" disease of Dr. Pym, for the benefit of the public. In truth, as it was

* Some there are, no doubt, born with an ungovernable propensity to say witty things *ad captandum*, and at all hazards, forgetting the wise warning, "Trust me, Yorick, this unwary pleasantry of thine will one day—."

† In further corroboration of what I have before said on this point, I beg to believed, at the time, that Dr. Barry had come out to Gibraltar with the intention of conforming to the more vulgar custom of seeing a disease before he would venture to write upon it, all the gentlemen in charge of hospitals, were, I say, quite at a loss to account for Dr. Barry's non-appearance, for several days, at their hospitals; but the mystery was satisfactorily cleared up when it was discovered that, moved by the same laudable zeal known to have influenced him on certain other occasions, he was, most usefully, employed in endeavouring to be *early* in the field in giving all possible information to the public on a subject involved in such obscurity. Let my readers just take the trouble to refer to the letter (p. 392.) which he wrote, about that time, to the gentleman at the head of his department in Gibraltar, (giving himself, by the way, the title of physician to the forces, when but staff surgeon, which he continued to be for exactly twelve months after,) observe, how in the little note subsequently added, for the emergency, to that letter, which is dated the 14th of November, 1828, he makes a desperate effort, by telling us that he uses the word *infectious* as synonymous with *contagious*, to explain away what he had said about the accumulation of "infectious disease" in a particular locality. But, strange to say, the Doctor has not given the public the benefit of the whole of his quote the following copy of a statement which I have received from Gibraltar, since I wrote the first part of this paper; namely:

"I, the undersigned, declare, upon my honour, that I was present at the mess of the 12th regiment of foot, on the Neutral Ground, about the middle of November, 1828, or soon after Dr. Barry's arrival in Gibraltar; when he, Dr. Barry, stated to the commanding officer, and others present, his unqualified conviction of the local origin of the fever then prevailing within the walls of the garrison; and that he had been led to this conclusion, after having made a topographical survey of the rock and its environs, the peculiarities of which he took great trouble to explain, and illustrated his arguments by a diagram, drawn by himself while sitting at the mess table: that Dr. Barry, further, expressed his anxious desire for the arrival of Dr. Chervin from Paris, as he thought their united researches would tend to throw great light on the formidable disease.

"I further declare that, soon after the arrival of Dr. Pym in the garrison of Gibraltar, I heard it reported that Dr. Barry had become an advocate for the importation of the fever alluded to from some foreign source; and this sudden change of opinion was often the subject of conversation afterwards among the officers of the 12th regiment, and several of them, as well as myself, expressed surprise, as it appeared to us this could not be the result of mature investigation, as the Doctor had been so short a time among us on the Neutral Ground. In conclusion, I appeal to the officers then present for the truth of this statement.

(Signed) "JOHN GALLICE,
"Assistant Surgeon, 12th Regt. of Foot."

*Gibraltar; February 10th,* 1831.

elaborate illustrations on this occasion: a copy of the original of the production in question, taken from the medical office at Gibraltar, is now before me, in which I observe he goes on to explain to his official chief (Dr. Broadfoot,) what the most thorough non-contagionist will not feel disposed to dispute with him. In continuation of what he has already given us of the letter in question, he proceeds to express himself thus: "The Naval Hospital is, as you know, a hollow square of houses built in a well," (built in a *well!*) "where the air is never refreshed nor even agitated, except by a kind of *tornado* or flurry, during the whole summer and autumn. I have no hesitation in giving it as my opinion that it has been a most efficient source of death and infection during the whole progress of the present epidemic, and still continues to be so, instead of affording any alleviation of either of these scourges. I mean to visit the hospitals of the south this forenoon, and to take with me some ounces of the chloride of lime, to be used as a disinfecting agent, if you have no objection. You are at liberty to submit this letter to his excellency, if you think proper!!" We have already seen that the Doctor has shewn himself not over and above particular in clipping off, and adding to, documents: he concludes this letter, which he has so partially given to the public, with the following postscript: "I shall very shortly submit a more detailed statement of my opinions on the subject of the fever to you. D.B." Perhaps Dr. Barry here alluded to his *local origin paper.*

I am ready to admit, that the same paramount consideration which prevented Dr. Barry, on his arrival at Gibraltar, from immediately seeking a knowledge of the disease at the established military and civil hospitals, as the gentlemen of the French commission did, prevented him from soiling his fingers, on any one occasion, at the post-mortem examinations, after his arrival at Gibraltar: this, people, who were not aware of his having been otherwise occupied, thought passing strange, from its having been particularly expected that he, as staff surgeon, would have taken an active part in those important and interesting examinations. Not disposed, then, to say that his absence was without a reasonable cause, I am bound to state, lest the public should be led into error on important points, by the supposition that he is, from his acquaintance with the disease, an authority to be relied upon in questions relative to the nature, &c. of yellow fever, that I can confidently call upon every medical gentleman in charge of an hospital, on Dr. Barry's arrival, for a corroboration of my former statement; and as he has mentioned the gentlemen of the French commission, I here beg leave to present a translation of a letter from one of those gentlemen, in support of my assertion.

"I send you, my dear Mr. Fraser, what I have to say relative to the two points on which Dr. Barry found it necessary to quote my name. It is assuredly not my intention, in this letter, to expose the errors to be found in the rest of his document," (Rejoinder:) "I leave them in your hands, and you will, I trust, do them prompt and ample justice. Notwithstanding, I cannot close my letter without expressing to you my admiration of all the talent displayed by our able colleague (*confrère*) in endeavouring to bring into light his labours during the late epidemic at Gibraltar. He tells us, for example, speaking of his researches in pathological anatomy, 'I witnessed autopsies, with Dr. Pym and others; at the military hospital, with my colleagues of the Anglo-French commission; at my own hospital I missed none, and all the fatal cases were opened there. I took notes, on the spot, of the living and dead.' For my part, with the exception of about three or four, I assisted at all the post-mortem examinations made at Gibraltar from the 24th of November, 1828, to the end of the epidemic, and I can with truth say, that Monsieur Barry honoured us with his presence very rarely (*fort rarement*) either at the civil hospital or at the military hospital, where the principal part of the examinations took place. I can affirm besides, that I do not recollect to have seen him in one single instance 'take notes, on the spot, of the dead,' but I recollect quite well to have heard him, several times, ask Monsieur Louis for his notes.

"In regard to the rest, on our arrival at Gibraltar'' (French commission,) "the surgeons of the Military Hospital informed us that they had not as yet had the pleasure of seeing Dr. Barry at that establishment; and, during the epidemic afterwards, I do not recollect to have once (*une seule fois*) seen him in the wards of the military hospitals; whether at the hospital called Naval, the Artillery Hospital, or the hospital at Windmill hill. His observations were therefore confined, nearly, to the hospital at North front;" (outside the walls) "where there was but a very small number of characterized cases of yellow fever, (*ou il n'y'eut qu'un fort petit nombre de malades de la fievre jaune caracterisé,*) a circumstance which might have contributed to lead Dr. Barry into error regarding the pretended wonderful effects of the remedies (*pretendus effets merveilleux de moyens therapeutiques,*) which he employed at that little establishment. 'Thus, he told me one day, with much enthusiasm, that he cured twelve out of fifteen patients. I replied, that, if he had arrived at Gibraltar six weeks sooner, he might very well, out of fifteen, have lost twelve.

"Your devoted servant and friend,

(Signed) "—— CHERVIN, D.M.P."

"*Paris;* 10*th December*, 1831."

"To Mr. Hugh Fraser."

Can evidence like this, from a person of such high character, be for a moment, resisted?

The statements in the above document can, if necessary, be corroborated by several other gentlemen; from some of whom, as well as from M. Louis, Dr. Barry wished to have notes of autopsies; but, as he had declined partaking of their labours, and aware of his wish to appear in public as an authority at this cheap rate, they declined giving them. Dr. B. exclaims, cavalierly enough, "But why descend to talk of these matters?" The reason is obvious enough, Dr. Barry, however unpleasant to you they may be. He says, that he witnessed autopsies at the Civil Hospital, with Dr. Pym and others: I have some recollection of his having been at the door of my dissecting house *one day* with Dr. Pym, but never did he enter to aid in any examinations going on; and it is to the knowledge of several, who thought it at the time very strange, that, at the little establishment near camp, to which he alludes (p. 399), his visits to that place, where a *very few* cases were opened, were more or less of the same casual description: I have no hesitation in maintaining that those visits consisted of inquiries at or just inside the door, as to what others were doing, or a request that Assistant-surgeon Moore, or somebody else, would send him the result of the examinations. What is now to be thought of Dr. Barry's appropriating to himself, which he evidently wishes to do, the observation, which nobody will be disposed to dispute, as to "a few cases diligently observed and carefully noted at the moment are worth a cartload of confused recollections."

If Dr. Barry was not content with the very reasonable excuses which others were ready to plead for his not going, on his arrival at Gibraltar, to the established hospitals, military or civil, where cases of the disease were to be seen, surely he could not reasonably expect people to listen to him very gravely, when he tells them, (p. 393,) "I saw and conversed with most of the civil practitioners." Shades of Hippocrates and Sydenham! not go, then, to study the disease at the bedside of the sick, on his arrival at Gibraltar! No, he does not venture to say any thing of the kind: he thought it best, as I have said, for him to fulfil what he calls in his Code Sanitaire his "mission," by writing about it first. Well, he says, he conversed about matters with civil practitioners, and made himself acquainted with the different modes of practice, civil and military: with one practitioner, in particular, I am well aware he had frequent conversations, a Dr. Mateo, who had charge of a certain number of tents on the Neutral Ground, designed as a casualty hospital for the use of the encamped civilians of the poorer class. This establishment, which was situated at the part of the civil camp farthest from the rock, was not appropriated, at any time, for epidemic cases; so that, were Dr. Mateo ever so well disposed to use active remedies, he had no opportunity of doing so, on the occasion in question, his cases not needing them. It was quite unfair, then, of Dr. Barry to hold this person's practice up, in making a comparison (as I am quite aware he did, from his communications on the subject to Mr. Wilson,) unjustly towards those whom he has since called his colleagues, and unjustly towards the profession; while he kept out of sight the practice of the very intelligent and experienced civil practitioner, Dr. Ardevol, who was, I believe, acquainted with all that had ever been done, in Spain, regarding the treatment of yellow fever, and who, having the bedside treatment of the yellow-fever lazaretto tents for civilians in camp, considered as his sheet-anchor that very remedy (mercury, whether by inunction or otherwise,) which the speculative secretary *decried publicly* as the cause of greater mortality among the military; and the practice of Dr. Bobadilla, acquainted with the epidemics which have occurred in Andalusia for the last thirty-five years, who, on the appearance of that of 1828, proposed to forward a memorial to the lieutenant-governor, to be allowed to have a certain number of persons placed in an hospital, under his special care, in order that he might adopt the plan which he had found particularly successful; namely, mercury, chiefly by inunction. Drs. Ardevol and Mateo were, I believe, the only medical men who had, outside the walls, where Dr. Barry lived, charge (by which I would always beg to mean contrary to what Dr. Barry's application of the term might be,) of the bedside treatment of civilian sick in hospitals. Within the walls, Dr. Barry might have found, on his arrival, that Mr. Wilson and myself were in charge of the Civil Hospital, in which some hundred cases had been received during the epidemic; and, had he consulted me, or come to my hospital, I could have explained to him that we were certainly far from being particularly fortunate, although various plans were tried which had been recommended by authors of repute: indeed, at one time, during the early part of the epidemic, we were more unfortunate, I believe, than our friends of the army; for the cases were of so severe a grade that all who entered, whether man, woman, or child, seemed fated to die; as an instance, of the first thirty-five Jews who were admitted, nearly every man died!* At that time a fatal issue was

* Numberless are the proofs on record of this wonderful disease destroying almost all, at one period of an epidemic, and becoming afterwards remarkably plainly denoted in the countenances of almost all who were received into the hospital: to expect advantage from any medicines seemed as hopeless as the application of remedies to a dead body. In the Ordnance and 43d regiments, a most severe form of the disease also appeared in the early part of the epidemic,' as was specially reported upon to the lieutenant governor, and to the head of the medical department in England, by the late Dr. Hennen: this circumstance is also recorded by Dr. Smith in his paper. We, who had the charge of civilians, had certainly nothing to boast of at the winding-up of the lists of mortality; for, according to Dr. Smith, who seems to have taken pains to obtain accurate results, we lost (between us all, civil practitioners,) 1281, out of 4701, including women and children; the loss of the military (including officers, women, and children,) having been, according to that gentleman, 512, out of 2002 attacked. By what is recorded of the usual progress of this fever, the soldiers being of more robust constitutions, and brought up in colder latitudes, might have been expected to have suffered more. Another cause which might be supposed to influence the average mortality greatly against the military, in the winding up of numbers on such an occasion, would be that the *number* of *women and children liable to be attacked* is vastly greater among the civilians, and, in them, the disease *usually* passes off with more mildness; for, although I have said that, at one time, the disease was very severe among women and children, this was not remarkably the case afterwards; nor was it the case after that period, generally throughout the town and territory: for instance, in the practice of one foreign medical man, (a Mr. Dias,) who made a distinction, in his morning states of sick, between men, women and children, we find that, of seven men whom he treated, between the 3d of November and the 27th of December, *three* deaths took place; while, of eleven women and children attacked, *only one* died. Had I in reality, as I believed I had at one time, cause to suppose that the disease was less fatal among the civilians, in my hospital, than among some of the corps in the garrison, the circumstances just mentioned offered a simple explanation. Such facts as these must be placed on record, for two reasons: first, because a cabal was most foully and unworthily raised against the practice of mild. M. Rochoux, one of the French commission to Barcelona in the epidemic of 1821, notices this particularly in his work, where he says that, on the first appearance of the disease, the loss was as nineteen out of twenty; in the middle much less, and at the close only two out of three! He observes, that the vulgar are apt to believe that the extraordinary mortality, on such occasions, proceeds from the remedies employed.

experienced men, who were perfectly masters of whatever had been said in favor or against the treatment of yellow fever in every part of the world, which, to the indignation of every honourable mind, drew on those men calumnies and injuries of the most iniquitous kind; in the next place, because it would be nothing short of crime to suffer the incompetent authority of my antagonist to pass current, and perhaps hereafter more or less prejudice inexperienced people against one of our greatest resources in a terrific malady.* Here, then, are reasons which, I presume, will be considered amply sufficient to warrant my entering on details likely to place the occurrences at Gibraltar touching this part of the subject *fairly* before the public: most gladly would I now, were it possible, admit that the manner in which they have been laid before the public, by certain persons, was owing to inadvertence, or any other excusable cause. Here, as in other instances, we find, indeed, the utmost art employed to make out a case favorable to particular views.

Over and over again has the truth respecting yellow fever been asserted, that, in its mild form, patients will do well without any remedies whatever; while, in its very worst forms, remedies cannot be looked to as of any use: between these two grades there is a third, of great danger, but in which remedies, good nursing, &c., may be expected to carry the patients through, especially if they are seen soon after the commencement of the attack. Now, in this last form, it is that more can be said of mercury, in one form or another, as a remedy, than can be said of any means whatever hitherto practised: that its exhibition will, in the grade of fever in which most is to be expected from it, often prove unavailing, is most true, if patients have not applied soon enough, or the numbers become so great at the explosion of an epidemic, that measures for its due exhibition cannot possibly be carried into execution, or, from idiosyncrasies, the remedy does not make its ordinary specific impression, indicated by the flow from the salivary glands: but if any fact in medicine can be said to be proved,

* What seems quite curious is, that, while so loud against mercury in yellow fever, Dr. Barry should seem to be unacquainted with the fact that no author is so full of details in proof of its efficacy, where good is to be expected from any thing, as his friend Dr. Pym; who tells us, in addition to many other facts, that, when at Carthagena, casually, during an epidemic of yellow fever, and having been asked his opinion by the Spanish medical men, recommended it in *large* doses: his faith in it, at Gibraltar, does not seem to have been much shaken, when we know that, in consultation with the surgeon of the Ordnance, he recommended it to be given, in repeated doses, to the commanding officer of artillery.

it is that the system, once fairly affected by that medicine, so as to have the effect of producing a discharge from the salivary glands, the patients, at least in the proportion of thirty-nine out of forty, may be considered as safe. This has been authenticated by numberless practitioners, from the days of Rush and Chisholm up to the Gibraltar epidemic of 1828, inclusive. Another thing, which seems to be well proved, is that, under other treatment, though numbers will do well, an experienced person will tell you that, in this most incomprehensible disease, he never feels confident as to his patients' safety till perfect health is established; it being of very common occurrence to find a patient at one visit free from all alarming symptoms, and in every way promising to do well, while, in two hours afterwards, he will be found ejecting black fluid from the stomach, and in the agonies of death. This is what never occurs, as far as I have seen or heard, where the salivary glands are affected by mercury: as far as the experience of 1828 at Gibraltar went, I may, I think, say, with certainty, that it *never* occurred in the practice of many gentlemen with whom I was intimate. That relapses sometimes occurred after convalescence had been established, in cases where the system had been affected, there is, I believe, no reason to doubt; but I think in a less proportion than when this had not been the case.

Holding in view the circumstances just stated, enlightened men of great experience in various parts of the world, have, though for a time, perhaps, speculating on more success from other remedies, recurred to the mercurial system, as the best thing to be done, in a disease in which they admit that *no practice is satisfactory*, if the grade be very severe. As to what has been sometimes advanced against the exhibition of mercury, "Oh, you are losing time by trying to get the system affected," I will admit this to be of some validity, if, after an impartial examination of the history of the disease by writers, whether British, French, Spanish, or American, the smallest confidence is to be placed in any other remedy whatever. Those who enter deeply into the subject of yellow fever, know well that what occurs, in occasional mild epidemics, or even at certain periods of the same epidemic, regarding "great success" from the adoption of mild remedies, or of no remedies at all, would be found to be quite illusory, if applied to the disease under more ordinary circumstances. To little avail has a mild, or *placebo* system, which Dr. Barry, with his limited view of matters, has thought proper to speak about as the best, been adopted over and over again, in epidemics in Spain, by practitioners, in their want of confidence in the efficacy of any remedies: to little avail had some men, holding the first rank in the profession, adopted that system at Barcelona in 1821; to little avail has nearly the whole population of a district or town, in Spain, been confined to the employment (necessarily so, from the want of medical men,) of the ordinary remedies; their own domestic remedies, " mild, frequent, oily purgatives, with acid diluents," which Dr. Barry tells the College of Physicians and the public were so much more successful than others, according to the " therapeutic records of the Gibraltar epidemic:" to little avail have these things been done, for the whole attacked in those instances seemed doomed to extermination, the mortality having, according to records, been so enormous, as every body who writes on yellow fever *ought* to know, and which, if Dr. Barry knew when he wrote his paper for the College, or when he assisted so openly in raising a *cabal* at Gibraltar against his "*colleagues*" *(!)* for using an active remedy, he was, in more ways than one, criminal in concealing. I think it will be presently seen that I am not employing *unnecessary* severity, when I assert that Dr. Barry, while making out (p. 392, 3) a case calculated to cover him with glory, and sink his " colleagues," below the ordinary range, has made use of nothing short of deception. The cause of truth, no less than self-vindication from this gentleman's attacks, obliged several gentlemen at Gibraltar to examine, closely, all the management which he had recourse to in his endeavours to shew their utter incompetence, as compared to himself, in the performance of their duties; and the following is (agreeable to undoubted information procured by me at Gibraltar, or transmitted to me since,) the true state of matters. I may reckon, I think, upon the attention of your readers to the details, if it be recollected that (though denied by Dr. Smith as to their general accuracy,) the circumstance which Dr. Barry adduces, in support of his statements, on points so important to nations, remain on record unrefuted.

In explanation of the occurrences at what Dr. Barry calls *his* hospital, (sheds erected outside the walls, at the recommendation of the chief medical officer, about the end of the epidemic season, as a branch hospital, for the reception of the fresh cases of three regiments encamped on the Neutral ground, and which by no means placed him, as the asserts, "in charge of the sick of three out of the seven regiments of which the garrison consisted,)" it did happen that one of the three surgeons (43d), whose newly attacked cases had been put into this branch-hospital, on finding, from the chief medical officer, that *he* was considered as the responsible person for their treatment, took charge of them accordingly, in five or six days after the opening of this establishment; the main bulk of his patients at the principal hospital, being at the time convalescent, were looked after by one of the assistant surgeons. At p. 392, Dr. Barry alludes to "official" returns, (as he now calls them, though at Gibraltar he made a solemn assertion, in public, that they were only returns given him for his *private* use,) by which he would make it appear that the sick of the regiment in question were under *his* care for the period he points out. Now, it does seem quite surprising how Dr. Barry can venture to make such statements, when it has been proved, in a manner which he surely can never forget, that the surgeon of the 43d regiment took charge of his hospital, in the branch or detachment hospital, from the period just pointed out to the end of the epidemic season, prescribing for his patients, and directing the use, where he judged necessary, in the few cases that required it, of that very remedy which Dr. B. says *he* suspended the use of *in toto:* all this was clearly shewn to have been the case, at Gibraltar, by registers, and by other proofs, not requisite for me to enter upon here. It also appears, by an authentic copy in my possession of the identical return or list which Dr. Barry quotes, that of the three or four cases of the 43d regiment which were at all seriously ill, under Dr. Barry's care for the first few days, and to whom not one particle of mercury had been given, two died: William Fisher, admitted 14th November; John Kelly, admitted 15th November, 1828; and that, in the same corps and hospital, of four, to whom mercury had been given* in a more or less quantity, one only died. It appears, by a copy in my possession, of another of the documents from which Dr. Barry draws his statement now under consideration, that, in the 42d regiment, the only death (Robert Campbell,) in the period specified by Dr. Barry, was where not a grain of mercury was given. Dr. Barry, seeing how things were going on against his experiment-making, asked for the returns of sick, *at the end of a month*; that is, from the 15th November to 14th December, when he formally told the then surgeon of the 42d, who had but lately arrived in Gibraltar, that he should not take any farther share in the management of the cases; but a man of this regiment, admitted on the 15th, died on the 18th of December, so that *he* does not of course, appear on Dr. Barry's lists; and it will be seen, by a document which I shall produce from the surgeon of this corps, what little reason he had to be satisfied with the whole result. Regarding the sick of the 12th regiment, as noticed by Dr. Barry, I have the authority of the surgeon for stating that he considers the statement

* Sow, Fell, Clanay, and Robinson.

made by Dr. B. as very unfaithful: he was himself unable, from the state of his sick in his main hospital, to assume the responsibility of those in the detachment hospital, but saw the patients frequently; and I have his authority for declaring that, of the whole twenty-two men admitted as yellow fever cases, according to Dr. Barry's lists, *very few of them indeed* ought to have been at all classed under that head; and that, of those few, the little success was very remarkable, four having died. Dr. Barry may say, "Oh, I have signatures to prove that the whole were yellow fever cases." No, he has no signatures to returns proving this; for I have in my possession the original copy of the return of the 42d regiment, from which those of the two other regiments were framed, and by that it appears that the heading of the document, as drawn up by Dr. Barry himself, or by somebody under his direction, ran thus: "*Return of men treated* in the regimental hospital of the 42d regiment at the sheds, from the 15th November, to the 14th December, 1828, shewing the period of convalescence of each, and the mode of treatment adopted." Many of the patients, especially on the first establishment of this hospital, were selected for admission by Dr. Barry himself, and the fact of fifty-eight patients at the close of our epidemic, having been treated in a particular building will be scarcely received as an evidence of their having been cases of yellow fever; more especially when, in addition to what appears from Dr. Chervin's letter, and the assertion of the surgeon of the 12th regiment, it was also shewn at Gibraltar, that the surgeon of the 43d regiment, on taking charge of his sick, which I have already stated had been for five or six days under Dr. Barry's care, felt it necessary to strike out of the list of yellow fever cases the names of three men respecting whom the error was most glaring, privates Cosbrooke, Clemo, and Gaining; and yet, though we find these men not entered as yellow fever cases in the official returns of the regiment for the period, sent to the Medical-board office, they are to be seen among the names on Dr. Barry's list; where, according to him, they stand as examples of his superior management of a disease as baffling as the plague. In the eleven cases of the 42d regiment brought forward, it would appear that as little precision had been observed as in the other two regiments, if we can judge from one very remarkable case of dysentery in its purest form, as noticed by several who had seen the man (William Watt), and whose case is called by its proper name in the medical register of the regiment, as appears by the following certificate of the gentleman who is now surgeon of that corps, viz.

"I find, from the register, that William Watt, third company 42d Royal Highlanders, was admitted, with dysentery, by Mr. Macleod into the North front hospital, on the 24th of November, 1828, and discharged on the 2d of December following.

(Signed) "B. NICHOLSON,
"Surgeon, 42d Royal Highlanders."

"*Gibraltar; May* 14, 1830."

With respect to convalescence after mercury, I believe that were it necessary, those whose testimony might really be of any value, could come forward in perfect contradiction of Dr. Barry's statement, by proving that numbers of the cases of the most favorable re-establishment to health, were those who had had that remedy administered.

One more point now only remains to be noticed on this subject, so important that the public should be rightly informed about: Dr. Barry talks of having declined the use of mercury in "consultation with my colleagues of the Sheds' hospital," without, however, saying who those "colleagues" were: he also stated, *on a certain recorded proceeding* at Gibraltar, that, in corroboration of his own opinion as to the exhibition of mercury in any shape being prejudicial in yellow fever, Mr. Macleod of the 42d, "who has been thirty years in the service, concurred, as did nearly every medical officer who has been under my direction." What is to be thought of such assertions, in the face of the following certificates? the first coming from the gentleman whom he names; the others from those who, by possibility, may be supposed to have been meant by Dr. Barry.

"I hereby declare, 1st. That, though a very long time in his Majesty's service, I had never till last year, at Gibraltar, seen any case of the disease known by the name of the yellow fever. 2dly. That I did not arrive at Gibraltar, from leave of absence, till about the close of the epidemic, when a few cases only occurred in the 42d, and of which a considerable proportion unfortunately proved fatal under the *mild* treatment. 3dly. That, having had no experience of the effects of mercury, I could not, and did not, at any time state, 'the exhibition of it, in any shape, during the late epidemic, to be extremely prejudicial.'

(Signed) "S. MACLEOD,

"Surgeon, 42d Regiment."

"*Gibraltar; 17th March*, 1829."

"I beg to declare, 1st. That I have been thirty-four years in his Majesty's service. 2dly. That I have witnessed three epidemics in this garrison. 3dly. That, far from having stated, at any time, that the exhibition of mercury was prejudicial, in the late epidemic, I found, after great experience, that it was the only remedy to be relied on, and in a manner our sheet-anchor, in the disease known by the name of yellow fever, such as it has appeared at Gibraltar, as an epidemic, on various occasions. I am further borne out in this opinion by the official report of Dr. Gilpin, principal medical officer during the epidemic of 1813, in this garrison, and which report may now be seen in the Medical Office. In that report it is stated, that the medical officers were agreed as to the superior efficacy of the mercurial treatment, as was Dr. Gilpin himself, after extensive experience during a long residence in the West Indies.

(Signed) "R. AMIEL,
"Surgeon, 12th Regiment."

"*Gibraltar; 20th March*, 1829.

"I have no hesitation in declaring, 1st. That, instead of declining to exhibit mercury during the late epidemic, I made use of it, from a thorough conviction, grounded on experience, of its superior efficacy to all other remedies. 2dly. That, far from having expressed, at any time, that the effects of that remedy were prejudicial, I continued the use of it.

(Signed) "J. GILLICE,
"Assistant Surgeon, 12th Regiment."

"*Gibraltar; 16th March*, 1829,"

The profession has here, then, as on other points, ample evidence shewing how far matters relative to a very important subject had been fairly placed before the public.

I have now done with questions regarding which I cannot be supposed to have had any interest more than as a member of an honourable profession, and which people will probably be disposed to agree with my antagonist were perhaps brought forward "in an evil hour" for himself: between us, owing to your kindness in affording space for lengthened details, the profession is likely to obtain what is so much its due. In some of the details which I have felt it my painful but indispensable task to enter upon for the thorough clearing up of subjects of paramount importance, it may be perceived that the office has not been an enviable one. The cause to which I have been opposed, respecting the contagion, &c. of yellow fever, never could have required (had it been a fair one,) the aids which I have exposed in the foregoing pages.

*Fort Pitt, Chatham.*

